# Recent Advances of GFRP Composite Cross Arms in Energy Transmission Tower: A Short Review on Design Improvements and Mechanical Properties

**DOI:** 10.3390/ma16072778

**Published:** 2023-03-30

**Authors:** Agusril Syamsir, Lee-Woen Ean, Muhammad Rizal Muhammad Asyraf, Abu Bakar Mohd Supian, Emrah Madenci, Yasin Onuralp Özkılıç, Ceyhun Aksoylu

**Affiliations:** 1 Civil Engineering Department, College of Engineering, Universiti Tenaga Nasional, Jalan IKRAM-UNITEN, Kajang 43000, Malaysia; 2 Institute of Energy Infrastructure (IEI), Universiti Tenaga Nasional, Jalan IKRAM-UNITEN, Kajang 43000, Malaysia; 3 Engineering Design Research Group (EDRG), Faculty Mechanical of Engineering, Universiti Teknologi Malaysia, Johor Bahru 81310, Malaysia; 4 Centre for Advanced Composite Materials (CACM), Universiti Teknologi Malaysia, Johor Bahru 81310, Malaysia; 5 Department of Civil Engineering, Necmettin Erbakan University, 42090 Konya, Turkey; 6 Department of Civil Engineering, Konya Technical University, 42090 Konya, Turkey

**Keywords:** pGFRP composites, cross arms, latticed transmission tower, energy infrastructure, structural improvements, mechanical properties

## Abstract

Currently, pultruded glass fibre-reinforced polymer (pGFRP) composites have been extensively applied as cross-arm structures in latticed transmission towers. These materials were chosen for their high strength-to-weight ratio and lightweight characteristics. Nevertheless, several researchers have discovered that several existing composite cross arms can decline in performance, which leads to composite failure due to creep, torsional movement, buckling, moisture, significant temperature change, and other environmental factors. This leads to the composite structure experiencing a reduced service life. To resolve this problem, several researchers have proposed to implement composite cross arms with sleeve installation, an addition of bracing systems, and the inclusion of pGFRP composite beams with the core structure in order to have a sustainable composite structure. The aforementioned improvements in these composite structures provide superior performance under mechanical duress by having better stiffness, superiority in flexural behaviour, enhanced energy absorption, and improved load-carrying capacity. Even though there is a deficiency in the previous literature on this matter, several established works on the enhancement of composite cross-arm structures and beams have been applied. Thus, this review articles delivers on a state-of-the-art review on the design improvement and mechanical properties of composite cross-arm structures in experimental and computational simulation approaches.

## 1. Introduction

An electrical pylon is a tower that functions to hold electrical transmission lines above the ground. In common practice, they are classified into two types of designs, which are monolithic steel tubes and latticed steel towers [1,2]. To construct a high-strength transmission tower with good structural integrity, the towers are divided into several components, such as the peak, boom, cage, tower body, and cross arm. The cross-arm component is mainly used to grasp and secure the power cables. Generally, the cross arm in a suspension lattice tower is made from high-strength materials such as steel, wood, and pultruded glass fibre-reinforced polymer (pGFRP) composite. These towers form network lines to transmit the electrical power from power generators to substations before going to consumers [3,4,5,6,7].

Creep is a time-dependent plastic deformation that arises due to constant stress, as well as being an effect of elevated temperatures. In this case, constant loading on the composite structure under long-term conditions would cause an increment in deformation known as the “creep phenomenon” [8,9,10,11]. Despite this, polymers behave like elastic solids, even though they display a similar viscoelastic behaviour. Extreme environment, high stress, or a long period of service can cause this phenomenon to occur [12,13,14].

The reliability and durability of structural components may be at risk due to the creep phenomena of fibre-reinforced polymer (FRP) composites [15,16,17]. In this point of view, the structural issues regarding the FRP composite can be resolved by understanding its long-term creep responses. The material would ultimately fail instantly and without any warning if creep occurred [18,19]. The durable performance of composite cross arms has been used to replace the wooden structure in suspension towers within transmission and distribution systems. The durable performance that composite cross arms offer is one of their advantages; they can also be used as cantilever beams for street light support structures due to their strength, durability, and lightweight properties [20,21]. This is because of the creep behaviour of pGFRP structures in transmission towers [13]. For instance, Beddu et al. [22] found that when subjected to long-term focused pressures, the initial creep response causes a modification in the size and shape of the cross arm. Finding the worries about the consequences of composite degradation that result in composite failure in this situation would help to minimise the composite structure. Therefore, creep behaviour is a major issue for material structures that have endured loads over an extended period of time [23,24].

The composite degradation might have an impact on the catastrophic collapse of architectural buildings. Extreme loading or the manufacturing process may cause the composite to degrade throughout the course of its lifetime [25,26]. This investigation could concentrate on the safe limit parameter of the cross-arm construction to give consistent safety criteria, such as a critical evaluation on the safe limit parameter of cross arm structure to develop a standard safety requirement [27,28,29,30]. The mechanical properties of a composite structure need to be characterised, and this can be conducted by experimentation and computational modelling. Recent studies have carried out a variety of experimental evaluations and tests employing either coupon-scale or full-scale structural tests. Computer simulations for composite cross arms have also been the subject of certain studies in early mitigation assessments.

Several methods and modifications to extend the service life of the composite cross-arm construction have been developed based on the data collected. Lamination order, structural enhancement, and hybrid composite structure are all covered in these improvement initiatives. As load-bearing components, biaxially oriented polymeric fibres and fillers inside the matrix, for instance, would interact with one another, having the effect of combining many elements into woven textiles [31,32,33]. For instance, Zaghloul et al. [34] discovered that surface-reinforced arranged composites have higher lifespan after being exposed to bending fatigue, as they have 61 times longer life than that of bulk-reinforced arranged composites at 56 MPa bending stress. Aside from textile engineering, the composite cross arm can be improved by adding supports to the structure. The employment of additive in pGFRP composites also can allow for improvement in its durability as well as mechanical performance [35,36,37,38]. Additionally, an improved cross arm can be improved by incorporating a core structure into the composite profile, such as a foam or honeycomb core. These initiatives dramatically alter the mechanical properties and functionality of transmission tower building structures [39,40,41,42].

The cost of constructing and maintaining a transmission tower’s cross arm will affect how much electricity is generated over the course of several decades [43,44]. Many investigations have found that the composite cross arm is considerably distorted under broken wire conditions as well as when used for lengthy periods. It must undergo design improvements in order to be more serviceable. Therefore, this review study aims to provide a state-of-the-art analysis of the problems and developments related to pGFRP composite cross arms. The findings of this study are meant to give researchers and engineers a useful viewpoint on how to comprehend the mechanical performance of pGFRP composite cross arms and related investigations. At the end of the study, the research outputs from this review are expected to set a baseline for mechanical profiling of GFRFP composite cross arms with several design solutions for structural and design engineers.

## 2. Lattice Transmission Tower

Transmission lines are made up of a network of power cables that are all hooked up to each other. This system connects 420 power substations with the power plants via 11,000 km of network lines in Peninsular Malaysia [20]. Transmission towers, usually referred to as electrical pylons, are used to carry these power cables. The electrical pylon is often a structure that lifts high-voltage cables and acts as a support beam for racks, transformers, and other high-voltage equipment [45,46]. The typical height of this tower is between 15 and 55 m [47,48]. According to this perspective, the largest transmission tower structure in Malaysia is 500 kV, while the medium-sized towers are 275 kV and 132 kV [43]. 

**Figure 1:**
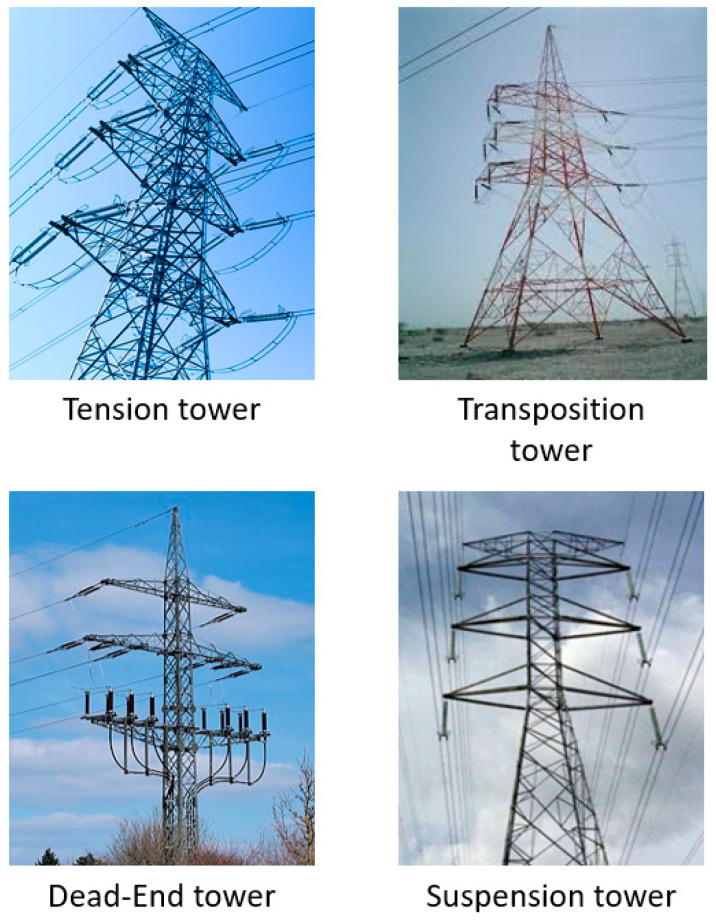
Types of latticed transmission tower [49,50].

The electrical grid’s transmission lines often have the tension, termination (dead end), suspension, and transposition (angle) towers arranged beside them (Figure 1). These four types of transmission towers are combined to carry the electrical supply [49,50]. A terminal tower, which connects the substation and generators to the tension tower, is the key component of the electrical grid [51,52,53].

As a result, the cross arm is made of this material to minimise costs while increasing electrical insulation in the surrounding region. Overall, the peak is applied to protect the earth wire attached to its tip and is placed at the top of the cross arm. Additionally, the cage holds the cross arm while being attached to the tower base via the tower body.

### 2.1. Cross-Arm Component

**Figure 2:**
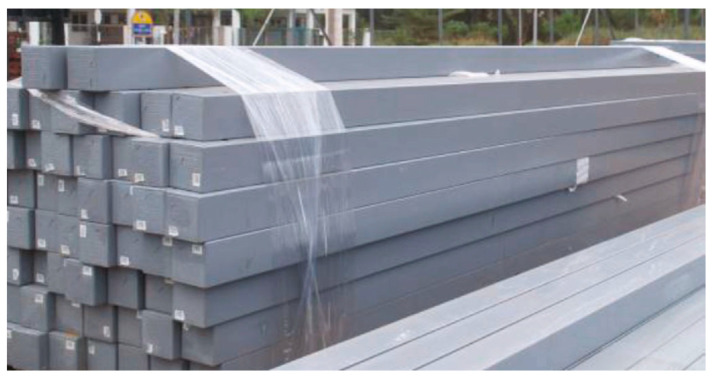
Cross-arm beams in stockpiles after pultrusion for distribution [57].

A cross arm is an extended structural component that is fastened to the structure’s end by galvanised sockets and fittings. To keep the utility wire with its insulators above a height from the ground, the structure is attached to the cage of the latticed tower by special fittings [54]. Depending on the location and cable size, the cross arm’s construction comes in a variety of sizes and shapes. The pGFRP composite and other nonconductive materials were used to create the cross-arm components [55,56]. Figure 2 displays the composite cross-arm stockpiles, which are ready to be installed in latticed transmission tower.

Earlier, the cross arms in transmission towers are made from wood due to the decline of wood as a timber source and the problems with wood in long-term applications [7,58]. Strong mechanical strength, electrical and thermal insulation, and good dielectric strength of the composites are recognised to set them apart [46,59]. A major factor in reducing the lifespan of the cross arms is creep, in addition to exposure to harsh environmental conditions and biological threats [60,61,62].

The necessity to employ composite as a primary material in cross-arm assembly has been prompted by a variety of circumstances, including the demand for cross arms with superior electrical properties and the challenges associated with hardwood cross arms [20]. Therefore, glass fibre-reinforced polymer composites are one of the best alternatives to hardwood cross arms in transmission towers.

### 2.2. Design Structure and Materials: Composite Cross Arm in Latticed Tower

**Figure 3:**
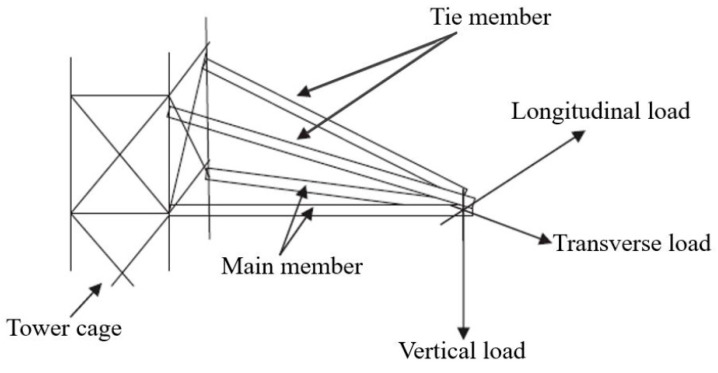
Location of cross arm in transmission tower with its forces acting on it [6].

The cross-arm components normally consist of two elements, namely the main and tie, that are connected by certain brackets in order to retain the cable in both vertical and transverse loads [63]. The cross arm’s configuration in the transmission tower and the forces acting on it are shown in Figure 3. In common practice, the square-profile composite of the cross arm is made from an E-glass fibre-reinforced unsaturated polyester (UPE) matrix via the pultrusion process. The common ratios are 37:63 to fabricate the composite beam with densities of 2580 and 1350 kg/m^3^ [22]. In terms of texture, the final product of the glass fibre composite has a fine surface and homogenous, unidirectional fibres along its matrix. 

According to Mohamad et al. [64], a composite cross-arm laminate is typically composed of various layers of varying thicknesses and orientations. The appropriate stacking sequence is essential to provide optimal mechanical performance and long-term resistance toward creep deformation. Asyraf et al. [13] studied five different stacking sequences of pGFRP composites on flexural and creep properties. They discovered that nine layers of 0°/45°/0°/−45°/0°/−45°/0°/45°/0° sequence are the fibre orientations for glass fibre composite cross arms to produce high flexure performance. These stacking sequence configurations seemed to be optimally manufactured in continuous roving fibre by alternating between 0° and ±45°. Regarding mechanical characteristics of the arm for the pultruded composite, the rupture and elasticity moduli are 29.8 GPa and 858.0 MPa, respectively [65,66]. This demonstrates that the pGFRP composite for the cross-arm application has outstanding mechanical performance. Table 1 displays the recent studies on the influence of stacking sequence of pGFRP composite cross arm on its mechanical and creep performance.

### 2.3. Manufacturing Processes of Composite Cross-Arm Beams

#### 2.3.1. Pultrusion

Pultrusion is the most popular technique for making composite cross-arm beams. It produces long, square-profiled structures by impregnating a unidirectional fibre with a thermosetting matrix [55]. The fabrication procedure features a production line that continually manufactures pultruded goods at a high volume rate, such as symmetrical profile beams [67].

**Figure 4:**
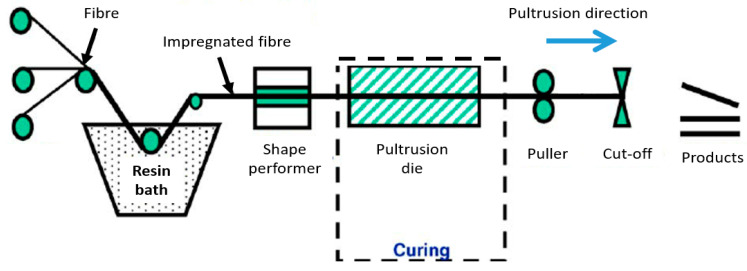
Schematic diagram of pultrusion process of pultruded composite [57].

In general, pultrusion is comprised of three distinct zones: pulling, heat, and pressure zones. To begin, a creel with the proper viscosity was used to impregnate the E-glass fibres with a thermosetting resin bath, such as unsaturated polyester. The pultrusion of a composite beam product is depicted in Figure 4. 

Throughout the process, ultraviolet rays are used along with thermocouple sensors, guide plates, and other components to guarantee the curing of impregnated fibres [68,69]. The guide plates help the fibre enter the resin bath during this operation so it may be impregnated. Two parts of the heated die (curing zone) gel at low temperatures and cure at high temperatures. The gelation point is the name given to this transitional state, which later solidifies to create a firm pultruded profile [70,71]. A heater that has a thermocouple sensor built into it raises the temperature of the die to a level that is suitable for curing.

A control system that can be easily monitored and adjusted on the monitor screen was used to regulate the pulling speed. To prevent scratching and damage during the process, a rubber grip was put on the hydraulic clamp to safeguard composite profiles (Figure 4). Pneumatic control and a hydraulic clamp were used to pull the profiles through the cutter.

#### 2.3.2. Filament Winding

The filament-winding technique can also be used to make composite cross arms; it applies constantly impregnated fibres with thermosetting resin comprehensively via a spinning mandrel. When fabricating the composite cross arm in this technique, numerous factors should be taken into account, including rotation number, winding patterns and angles, all of which are automatically controlled by a computer system [72,73].

**Figure 5:**
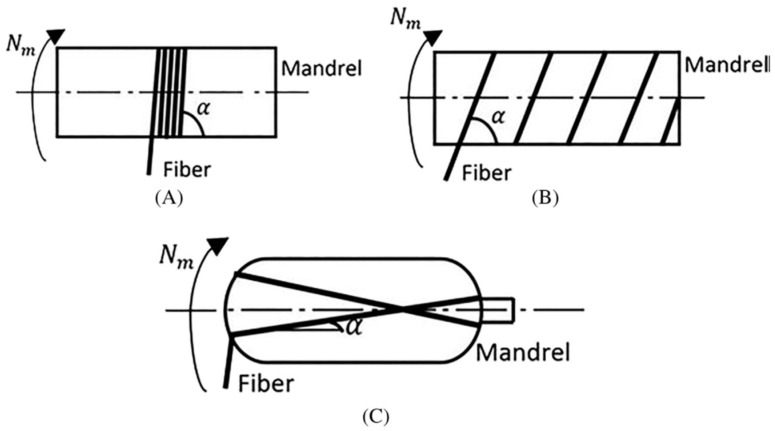
Types of winding patterns; (**A**) hoop; (**B**) helical; (**C**) polar windings [76].

Supian et al. [74], indicate that the ±70° filament-wound composite has improved energy absorption properties, which decreases composite structure failures. Better beams for cross-arm members in latticed towers can be made using this technique for better mechanical performance [75]. Hoop, helical, and polar windings are three major divisions of winding patterns, as shown in Figure 5 [76]. Morozof [77], asserts that the winding patterns of thin-walled composites affect how they behave mechanically. To ensure that the stress is distributed evenly in this situation, helical- and hoop-winding designs must be used.

## 3. Design and Structure Improvement

### 3.1. Influence of Mechanical and Load Bearing

Composite structures, such as glass fibre composites, have a high potential to experience crack propagation if the structure is not fully optimised during the design development stage. This phenomenon would be the basis for structural deterioration, as the composite structure experiences a deficiency of torsional resistance and load-carrying capacity [19,78].

Torsional–flexural strength and global buckling capacity are extremely reliable for global member bending, according to the study of Cardoso and Togahsi [79]. Delamination of composites would begin as a result of torsional reaction’s impact on the structure. In general, interlaminar damage and composite layer delamination may result in the delamination of composite structures. As a result of the structure being subjected to a high load intensity, delamination may occur in this instance at the interlaminar level of the fibre and matrix [80].

From the study by Cardoso and Togahsi [79], torsional–flexural strength and global buckling capacity are highly dependable to global member bending. Thus, the study of the general behaviour of buckling modes is influenced by torsional properties, which could benefit heavy structure applications. The influence of torsional reaction on composite structure would initiate the delamination process of composites. In this case, delamination can occur in the matrix at the interlaminar level of fibre and matrix as high-load-intensity implements on the structure [80]. In general, the delamination of composite structures may happen in the scale of interlaminar damage and delamination of composite layers. Subsequently, the composite structure may experience fibre breakage, kinking, and matrix cracking. Table 2 illustrates recent findings from previous literature on the influence of delamination on the degradation and shear properties of glass fibre composites.

Numerical and computational analyses have been used to describe the mechanical performance of a composite cross arm [83,84]. These include ones on the effects of laminate configuration on the failure characteristics of cross arms. A composite structural model was simulated in a different investigation by Turon et al. [85] to assess the delamination propagation under cyclic loads. Meanwhile, Selvaraj et al. [6] found that a postbuckling effect affected the properties of crushing, local buckling, and global buckling. They applied the cohesive zone model for quasi-static stress, which was derived from their experimental findings. In order to investigate the cross-arm failure, Selvaraj et al. [6] also carried out a mechanical performance test for composite cross arms and validated the outcomes with FE analysis. 

Additionally, the constitutive fatigue damage model used in structural analysis allows for the replication and validation of the research’s experimental findings. Cardoso et al. [86] developed a complete equation for pultruded composite square tube columns under concentric compression. Furthermore, the research found that the characteristics and interactions of crushing, local buckling, and global buckling were affected by a postbuckling effect. In this way, a composite cross arm beam’s square hollow portion is especially susceptible to buckling and failing under a heavy concentration load. Glass fibre composites that experience delamination would diminish their final loading capability, according to additional research conducted by Li and Li [87].

Consequently, postbuckling and other serious damage growth are the primary issues with thin-walled composite structures (severe deflection). The observation may have happened as a result of the buckling and slandering phenomena that occurred during its use. Understanding the torsion properties of composite structures is a crucial first step in preventing failures.

### 3.2. Creep Effect

**Figure 6:**
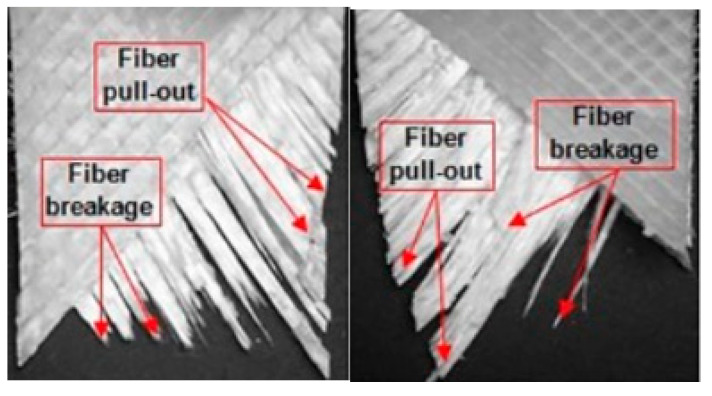
Post-effects of long-term loading due to creep failure of glass fibre-reinforced thermosetting polymer composites [88].

Creep is one of the factors that may lead to structural failure. In this instance, it is crucial to analyse the creep behaviour of a composite cross arm. The strengths of the material and the structure, failure initiation, elasticity, and viscoelasticity under constant load will be examined [19]. Overall, the understanding of creep is essential in the development of composite cross arms in order to resist the catastrophic damage of structure. For instance, Movahedi-Rad et al. [88] discovered that initial glass fibre-reinforced thermosetting polymer composite cracks start to occur during the primary stage of creep. Then, the crack propagation continues during the secondary creep stage, which causes the fibre to stretch and pull from its textile. At the tertiary creep stage, creep failure such as fibre breakage and pull-out (Figure 6) happened due to concentrated damage development at a high stress level. Several studies have developed a test facility to examine the creep behaviour of modern pultruded composite and conventional wood timber cross arms in coupon- [27,89] and full-scale tests [12,90]. The experimental efforts can be split into two categories: load-based (traditional) and temperature-based (accelerated). 

In a study by Asyraf et al. [27], the quasi-static and creep characteristics of wood timber and pGFRP composite cross arms were contrasted on a coupon scale. They discovered that pGFRP composites exhibit greater creep resistance than wood timber samples because they allow for higher stability throughout the transition from elastic to viscoelastic phases. Additionally, Asyraf et al. [12] investigated the effects of bracing a 132 kV pGFRP cross arm in a full-scale test, focusing on the creep behaviour using Findley and Burger models. The results thus show that the insertion of bracing arms increased the stiffness and structural integrity of the cross-arm construction.

Furthermore, the study by Asyraf et al. [47] developed the conceptual design of a cantilever beam creep test rig for a full-scale cross arm using a hybrid TRIZ–morphological chart–ANP technique. They also narrowed their work using finite element analysis to verify the strength and safety factor of the conceptual designs. In terms of creep test facility development, several test rigs were designed in order to cater to the study of the cross arm in an actual outdoor environment. Otherwise, Asyraf et al. also narrowed their work using finite element analysis to verify the strength and safety factor of the conceptual designs [48] together with a coupon test rig for flexural creep, specifically for cross-arm usage, using the same technique as the full-scale cross-arm test rig [61]. 

Therefore, to assure the safety limit for long-term applications, it is vital to conduct research on the creep properties of cross arms using experimental and computational analysis. Particularly for cross-arm constructions, the themes of creep strain, creep compliance, stress-independent material constant, creep failure, and creep life are fascinating.

### 3.3. Recent Studies and Related Works on Cross Arms

#### 3.3.1. Numerical Modelling and Simulations

**Figure 7:**
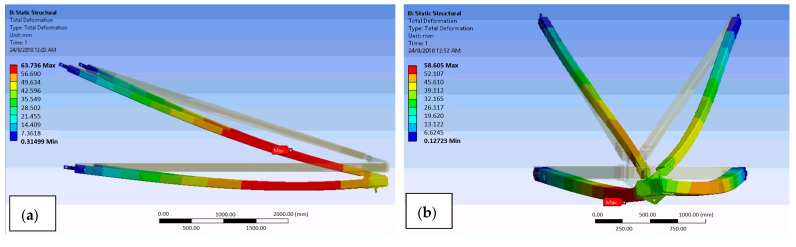
Deflection movement for (**a**) normal and (**b**) broken wire conditions [65].

Numerical analysis is a branch of theoretical analysis that predicts the behaviour of a composite structure based on theoretical inputs and conditions that have been developed. The numerical simulation technique can be used to address concerns regarding the sustainability of the current structure of pultruded composite cross-arm beams early on [91,92]. A numerical simulation was run by Al-Hayek et al. [65] to forecast the mechanical deflection, stress, and failure safety factor of a composite cross-arm assembly found in a transmission tower. The composite cross arm had the highest critical value at the outer ply in the major members in a broken wire state, according to the data. This resulted in the cross arm’s deflection, which is illustrated in Figure 7 by some lateral torsional buckling in the broken wire. The investigation came to the conclusion that a composite cross arm could experience delamination failure under a broken wire circumstance, as shown in Table 3.

**Figure 8:**
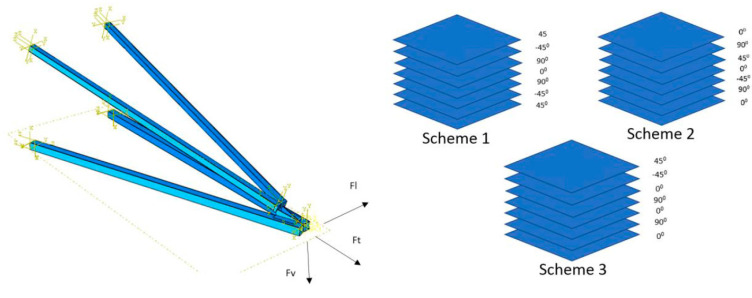
Schematic sequence of fabric layers of composite cross arms [83].

Mohamed et al. [83] investigated the influence of composite stacking sequence on the performance of a composite cross-arm structure subjected to multiaxial quasi-static loading. However, the fraction of layers that had distinct fibre directions had a substantial influence on the structure’s static displacement, as can be shown in Figure 8. Another study by Mohamed et al. [84] assessed the impact of laminate characteristics on the failure of composite cross-arm structures when subjected to multiaxial quasi-static pressure. In this investigation, three distinct lamination behaviours were used to analyse the deformation and failure characteristics of the composite cross arm. 

Otherwise, the stacking sequence did not have a significant impact on the static displacement of the structure. Nevertheless, both the stacking sequence and layer proportion with distinct fibre directions have demonstrated the production of failure in cross-arm laminate. The maximum deflection of composite cross arms with different fibre stacking sequences is shown in Table 4.

According to the discussion from the aforementioned, it can be concluded that the cross arm with the higher young modulus and ultimate bending loads only has one mode of failure, fibre buckling, which occurs at a deflection of 0.082 m under compression. However, the laminate designs and characteristics did not prevent the cross arm from failing when subjected to multiaxial loading.

#### 3.3.2. Coupon-Scale Analyses

Cross-arm development in latticed transmission towers has been studied at various scales. The coupon-scale test can also be used to carry out a forensic investigation. A crucial phase in characterising the material, which is exposed to real-world variables such loading capacity, temperature, and humidity, is the evaluation of coupon-scale tests.

The study by Syamsir et al. [93] conducted research on coupon quasi-static analysis on a glass fibre-reinforced polymer composite laminate which were affected by the fibre orientation (30°, 45°, and 90°), where the results concluded that the composite construction shows long-splitting failure modes along fibre orientations. Meanwhile, Asyraf et al. [27] used a coupon scale to compare the quasi-static and creep behaviours of wood and composite cross arms. The results show that the flexural strength of the glass fibre-reinforced polymer (GFRP) composite was significantly higher than that of the wood sample, whereas the GFRP sample showed high creep resistance and superior stability during the phases that change from elastic to viscoelastic.

**Figure 9:**
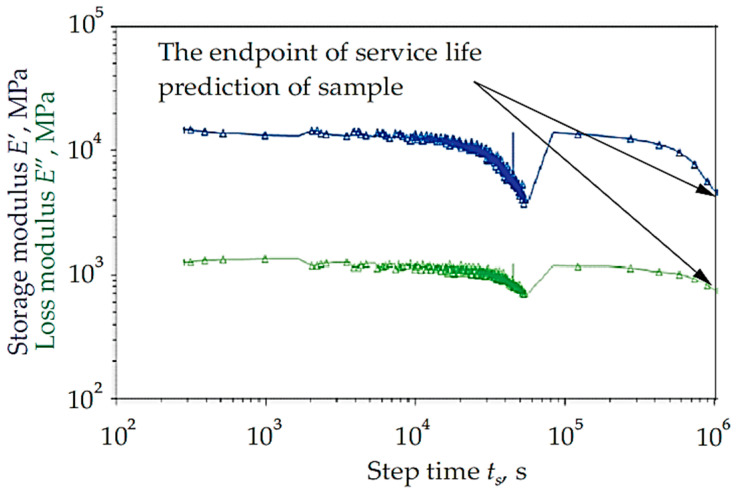
Master curve of both pGFRP composite samples at 95 °C, which indicates 25 years of service [94].

The creep properties of pultruded crosses in a transmission tower were studied by Johari et al. [94], who looked at the effects of calcium carbonate on these components. The study has evaluated their creep parameters using the time–temperature superposition (TTSP) concept and the conventional bending creep technique. As a result of the research findings, strain deflection, as shown in Figure 9, was generally predicted to have a service life of up to 25 years. Additionally, the method and material of calcium carbonate as an addition in this study would extend the useful life of cross-arm composites.

**Figure 10:**
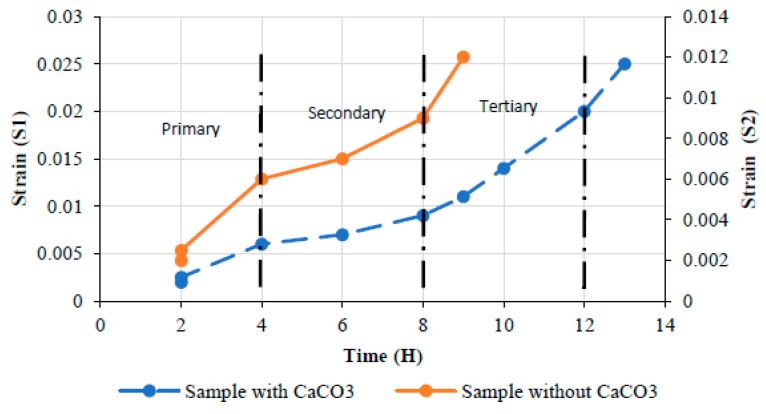
The influence of calcium carbonate as filler on creep properties of fiberglass composite [89].

On the other hand, Johari et al. [89] also conducted a short-term investigation of the creep performance of a fibreglass composite cross arm. The failure point for the calcium carbonate composite, which was achieved after 13 h of operation at 120 °C, was noted as needing additional time to deform. Therefore, adding calcium carbonate as filler to the composite would greatly enhance the laminate’s ability to resist long-term creep (Figure 10).

The numerical analysis results display that the most suitable creep model to forecast the creep pattern for both wood timber and pultruded composite samples was the Findley model, which is remarkable in having the least deviation from the experimental value. In this case, pultruded composite is the most promising candidate to be used in cross arms, since it is a highly durable material, while the Findley model is the most accurate model to analyse the creep properties of anisotropic materials.

#### 3.3.3. Full-Scale Structure Experiments

Cross-arm assembly side arms are exposed to high levels of humidity, temperature, and ultraviolet radiation [5,95]. These conditions hasten the ageing process of the structure and allow it to begin to fail [96,97,98]. A thorough analysis of these materials’ durability is absolutely necessary to address this issue [99,100]. Hence, full-scale structural tests on quasi-static and long-term creep must be carried out outside.

**Figure 11:**
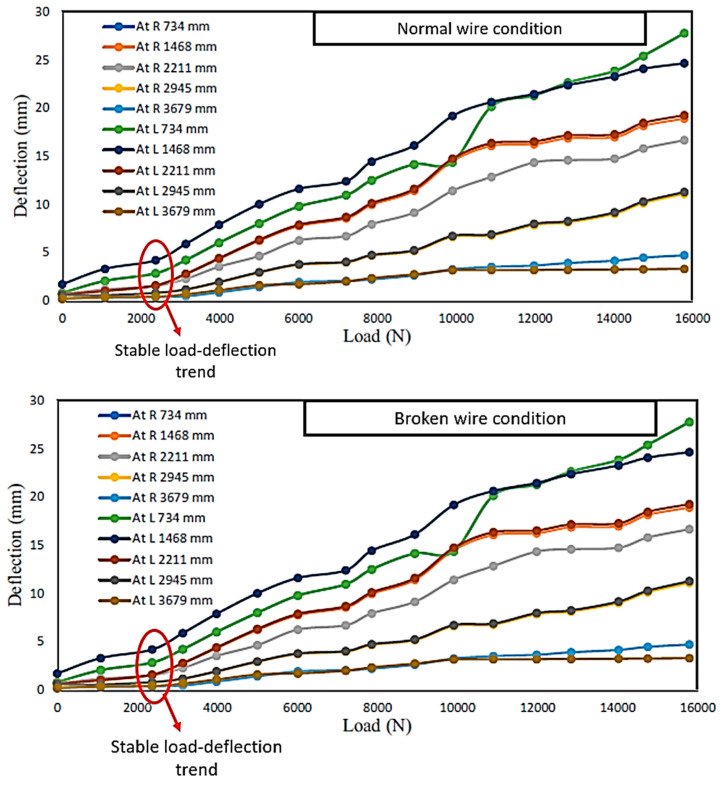
Load–deflection trends of normal and broken wire conditions of wooden cross arm in 132 kV transmission tower [101].

Sharaf et al. [101] performed a quasi-static analysis to determine how wire conditions affect wooden cross arms at 132 kV transmission towers. The study revealed that the wood cross arm can deflect under loads of up to 8000 kN, both under normal and damaged wire situations. Beyond this degree of load, the results begin to rapidly rise, which is caused by the beginning of microscopic-scale fibre breaking from shearing and buckling [102,103,104]. Meanwhile, the exaggerated data values for broken wire situations can be seen due to torsional irregularity caused by angularly focused loads acting on the cross-arm assembly [105]. Therefore, Figure 11 compares the load–deflection calculations of a wooden cross arm in a 132 kV transmission tower for normal and broken wire situations.

Furthermore, Asyraf et al. [90] analysed the effect of the bracing arms of wooden cross arm, as well as further studying the creep performance of a wooden cross-arm structure [12], discovering that the additional support from the bracing system would remarkably improve the creep resistance by reducing its strain during the 1000 h test. The addition of braced arms improved the elastic and viscoelastic moduli for long-term service applications. Following the aforementioned research, it was concluded that latticed towers with cross arms could benefit from the innovative creation of a multimethod strategy to evaluate and improve the service life of the cross-arm structure over the long term.

### 3.4. Advances Design of Composite Cross Arm

The failure issues of the composite cross-arm assembly in transmission towers have become essential issues [12]. These issues are related to the sustainability of current composite cross-arm designs. Sleeve installation, reinforcement with additional braced arms and incorporation of a core structure in the composite beams are being addressed. These efforts could potentially improve the structural integrity of the material without incurring a tremendous overall cost.

#### 3.4.1. Sleeve Installation

According to Asyraf et al. [12], composite cross-arm beams underwent substantial bending deformation at the middle main members. Regarding this matter, a probable failure or any crack propagation may be induced at this point, leading to structural collapse later on throughout its service period. In this circumstance, sleeves at major member beams for cross-arm structures have been suggested by a team of researchers led by Mohamad et al. [66]. ANSYS software was used to model the structural analysis of the composite cross arms. When subjected to multiaxial static loading, the alternative type of composite cross arms with a one-meter sleeve span fitted on both arms was examined for its effects on bending deformation.

**Figure 12:**
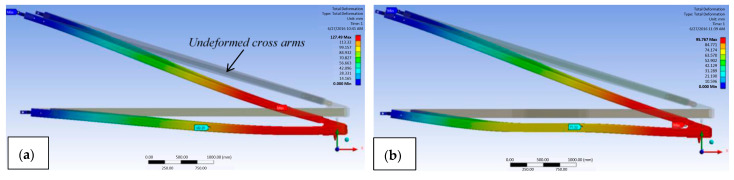
Deformation of (**a**) the current composite cross arm and (**b**) the sleeve-installed cross arm as subjected to multiaxial static loading [66].

Table 5 displays the comparison of deformation results for both the standard design and the sleeves with improved cross arms (Figure 12). In addition, the stress value of the improved cross arm seems to be lowered. In this manner, the sleeve can be classified as a practical answer to enhance composite cross arms’ structural integrity, which subsequently extends their life span with lower maintenance costs.

#### 3.4.2. Addition of Braced Members

To enhance the structural integrity of the cross arm, a composite structure can be applied with the incorporation of a bracing system. Most construction structures, such as buildings, bridges, and tunnels, incorporate bracing systems [106,107]. The retrofitting of braced arms as reinforcement in a structural system would function to restrain elastic buckling and improve external stiffness in its structure [108]. Braced frames and moment-resisting frames are commonly used in transmission towers and cross-arm assemblies. Due to lateral stresses from steel structures, these frames are an effective way to reduce deformation. The use of braced arms also helps the system to increase its axial compressive load-carrying capacity [109].

**Figure 13:**
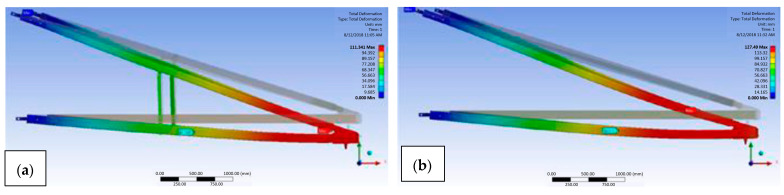
Comparison of simulated deformation experienced by cross arm (**a**) with and (**b**) without bracing [64].

Recent research carried out by Mohamad et al. [64] examined the use of two configurations of braced and current designs of composite cross arms in a 275 kV tower. They determined that the largest bending deformation that composites now suffer is 79 mm, but the addition of bracing would reduce this number to 63 mm. According to Figure 13, the composite cross arm’s total deformation was greatly reduced after the bracing system was installed.

**Figure 14:**
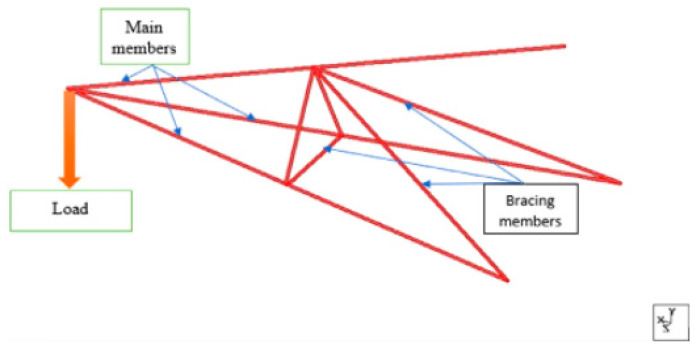
Final design of bracing cross arm in 132 kV transmission tower [110].

Furthermore, Sharaf et al. [110] developed an optimised bracing design for a cross-arm structure in a 132 kV tower, as shown in Figure 14. The work executed the concurrent engineering technique to generate several conceptual designs and optimise these designs using Skysiv software. The final selection of braced cross arms was implemented using an analytic network process (ANP). This conceptual design was selected because it exhibits the least deflection among all designs. Later on, Asyraf et al. [12] implemented the braced design proposed by Sharaf et al. [110] and discovered that the bracing system provided better durability and structural stability for long-term usage of composite cross arms. As a result, for most latticed transmission towers, early installation of bracing devices in cross-arm assembly can reduce maintenance work and the possibility for structural failure of the current installed cross arms.

#### 3.4.3. Incorporation of Core Structure in Composite Beam

Typically, the profiles of cross-arm beams would take the shape of a square hollow structure filled with white foam to prevent water from penetrating the beam. In several research studies, it has been recommended to use a lightweight core structure to improve structural performance and lengthen structural life.

**Figure 15:**
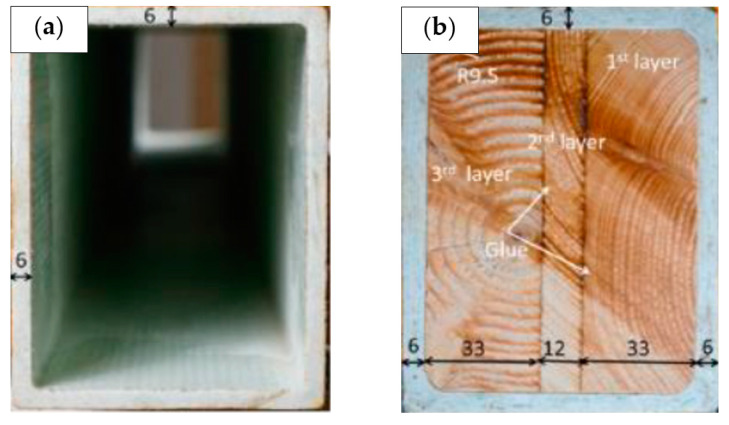
Specimens geometry and shaped for (**a**) hollow section and (**b**) sandwich section of pultruded GFRP beams [112].

To increase the overall strength of a square hollow beam structure, Qin and Wang [111] added a metal foam core sandwich. As the total deformation of local indentation decreases, the structural strength of the composite core structure is greatly increased. In the meantime, Zhang et al. [112] investigated a wood-cored glass fibre-reinforced polymer composite sandwich placed by pultrusion in another study (Figure 15). The investigation showed that the sandwich section offers substantial value in ductility and outstanding outcomes in load-carrying capability. This occurred as a result of the wood’s decreased capacity for plastic deformation under local compression along the sandwich bond and wood grain. Moreover, it was attributed to improved inner surface bonding between the wood core and the glass fibre composites. 

According to the aforementioned research, the use of core structures in composite cross arm beams has a great potential because they have better mechanical and structural loading capacities. Amir et al. [44] advised using a honeycomb core for the core structure since it has a high strength-to-weight ratio. Since the primary member beam experiences the greatest deformation relative to other points, they suggested only including the core structure there. Therefore, the incorporation of the core structure in cross-arm applications is highly possible, and it will assure the structure’s sustainability, which will result in extended serviceability and a reduction in cost.

### 3.5. Future Outlook for Newly Designed Composite Cross Arm

It is evident from the previous conclusions and observations that the glass fibre-reinforced polymer composite cross arm has a significant influence on transmission towers. This is because the material is less expensive, lighter in weight, and has good thermal and electrical insulation. Some researchers discovered that the construction starts to deteriorate and some parts of the cross arm eventually fail. In order to guarantee the cross arm’s longer life span, a modification to the construction must be made.

In order to increase the strength of a structure, cross arms with bracing are recommended by Sharaf et al. [110]. Moreover, Asyraf et al. [12,90] stated that bracing arms would uniformly distribute the loading force over the cross arm assembly. In regards, Mohamed et al. [66] proposed adding sleeves to the primary member beam of the cross-arm structure in order to prevent bending distortion. Otherwise, the results of the numerical study showed that the main member’s structural endurance when subjected to cable and insulator force is improved by the installation of sleeves. Hence, the feasibility of integrating a composite cross arm structure with the core structure was discussed by Amir et al. [44]. 

To demonstrate the improved cross arm’s performance in actual use cases, it is necessary to conduct a realistic analysis based on the recommendations for enhancement. Additionally, hygrothermal and hydrothermal test evaluations, respectively, can be used to counteract the effects of moisture at various temperatures on the cross arm [113,114,115]. Furthermore, numerous environmental elements, such as wind load, can also contribute to the failure of a structure [6]. Other environmental factors, such as exposure to fire and ultraviolet radiation, can also be responsible for a decline in the structural integrity of a building [15]. Thus, future investigations should consider coupon- and full-scale structure analyses and tests.

## 4. Conclusions

This review paper covers a state-of-the-art review of composite cross arms’ structural design improvements and mechanical qualities for transmission tower applications. Several studies have recently discovered that the composite cross arm has numerous advantages, including being lightweight and having a high strength-to-weight ratio. However, a number of studies have suggested that composite cross arms will eventually deteriorate and cause structural collapse. The conclusion remarked can be drawn below:

- A composite cross arm’s structural failure could be caused by buckling, torsional action or creep brought on by the application of multiaxial loading over an extended period of time.
- A number of thorough analyses of composite cross-arm constructions are being conducted in order to establish comprehensive views and understanding of cross arms, including quasi-static mechanical and creep experiments in full-scale and coupon-scale cross arms. For a comprehensive and analytical understanding, numerical analyses of the full-scale cross arm have also been reviewed in this study.
- Therefore, in order to address the concerns expressed in the aforementioned statement, several potential improvements to the current design of the cross-arm structure have been proposed. These include the addition of braced arms, the installation of sleeves, and the incorporation of core structures into composite beams.
- It is recommended that additional research be conducted in the near future to evaluate the aforementioned improved composite cross arms in a variety of conditions, including high moisture, surrounding temperature, and an acidic environment. This will ensure that the products can withstand extreme conditions and be used in transmission towers.

## Figures and Tables

**Table 1:** Fibre orientation and thickness of glass fibre composite cross arm.

pGFRP Composite Cross-Arm Fabric Orientation (°)	Ultimate Flexural Strength (MPa)	Strength Reduction Factor, χ(t)	References
45°/0°/45°	267.88	0.93	[13]
45°/−45°/90°/0°/45°	175.21	0.84	
45°/−45°/0°/90°/0°/90°/0°	355.96	0.93	
0°/45°/0°/−45°/0°/−45°/0°/45°/0°	436.29	0.95	
45°/−45°/0°/0°/0°/0°/0°/0°/−45°/45°	289.07	0.87	
±45°/90°/0°/±45°	242.60	0.87	[14]
±45°/0°/90°/0°/90°/0°	399.05	0.94	
45°/−45°/90°/0°/45°	421.35	-	[27]

**Table 2:** Degradation and shear properties of glass fibre composites in respect to creep.

Previous Literature	Time to Thermal Exposure (h)	Interlaminar Shear (MPa)	Degradation Depth (mm)	In-Plane Shear (MPa)
[81]	-	34.7 ± 4.5	-	53.7 ± 4.5
[82]	0	-	81.98 ± 8.60	-
	1000	-	51.97 ± 6.89	-
	4000	-	55.71 ± 5.16	-

**Table 3:** Failure safety factor for each layer in composite cross-arm main beam [65].

Loading Conditions	Fibre, s1	Matrix, s1	In-Plane Shear, s1 and s2	Out-of-Plane Shear, s1 and s3	Out-of-Plane Shear, s1 and s3	Delamination, s3
Normal	4	2.1	1.4	1.4	1.7	3
Broken wire	4	1.2	3.5	2	1.4	1

**Table 4:** Deflection of composite structure with various stacking sequence.

Scheme	Composite Laminate Lay-Up	Maximum Deflection, mm
1	[45°/−45°/90°/0°/90°/−45°/45°]	189
2	[0°/90°/45°/0°/−45°/90°/0°]	234
3	[45°/−45°/0°/90°/0°/90°/0°]	245

**Table 5:** Deformation results of both composite cross-arm configurations [66].

Configuration	Mid-Span Deformation (Mm)	Peak Deformation (Mm)
Current design	102.01	127.49
Sleeve-installed design	71.32	95.37
Percentage reduction with sleeve installation	30.09%	25.19%

## Data Availability

No data were used to support this study.

## References

[1] Prasad Rao N., Bala Gopal R., Rokade R.P., Mohan S.J. (2011). Analytical and experimental studies on 400 and 132kV steel transmission poles. Eng. Fail. Anal..

[2] Bantupalli R., Potireddy S., Baljai K.V.G.D., Santhosh Kumar B. (2020). Advantages of monopole transmission tower with new generation conductors. Int. J. Adv. Res. Eng. Technol..

[3] Awrangjeb M. (2019). Extraction of power line pylons and wires using airborne LiDAR data at different height levels. Remote Sens..

[4] Yu Y.Z., Ni J.K., Han J.Y., Ren B., Li A.A., Liu C.X. (2015). Influence of Overhead Ground Wire Height and Impulse Ground Resistance on Lightning Protection Performance of Distribution Line. Adv. Mater. Res..

[5] Zhu J.J., Schoenoff M.S. Effects of natural sunlight on fiberglass reinforced polymers for crossarms. Proceedings of the IEEE Rural Electric Power Conference (REPC).

[6] Selvaraj M., Kulkarni S., Babu R.R. (2013). Analysis and experimental testing of a built-up composite cross arm in a transmission line tower for mechanical performance. Compos. Struct..

[7] Rawi I.M., Ab Kadir M.Z.A. (2015). Investigation on the 132kV overhead lines lightning-related flashovers in Malaysia. International Symposium on Lightning Protection, XIII SIPDA.

[8] Asyraf M.R.M., Syamsir A., Bathich H., Itam Z., Supian A.B.M., Norhisham S., Nurazzi N.M., Khan T., Rashid M.Z.A. (2022). Effect of Fibre Layering Sequences on Flexural Creep Properties of Kenaf Fibre-reinforced Unsaturated Polyester Composite for Structural Applications. Fibers Polym..

[9] Asyraf M.R.M., Khan T., Syamsir A., Supian A.B.M. (2022). Synthetic and Natural Fiber-Reinforced Polymer Matrix Composites for Advanced Applications. Materials.

[10] Zhang Q., Zhang D., Lu W., Khan M.U., Xu H., Yi W., Lei H., Huo E., Qian M., Zhao Y. (2020). Production of high-density polyethylene biocomposites from rice husk biochar: Effects of varying pyrolysis temperature. Sci. Total Environ..

[11] Cardoso D.C.T., Harries K.A. (2019). A viscoelastic model for time-dependent behavior of pultruded GFRP. Constr. Build. Mater..

[12] Asyraf M.R.M., Ishak M.R., Sapuan S.M., Yidris N. (2021). Utilization of Bracing Arms as Additional Reinforcement in Pultruded Glass Fiber-Reinforced Polymer Composite Cross-Arms: Creep Experimental and Numerical Analyses. Polymers.

[13] Asyraf M.R.M., Syamsir A., Zahari N.M., Supian A.B.M., Usman F., Itam Z. (2022). Effect of Stacking Sequence on Long-Term Creep Performance of Pultruded GFRP Composites. Polymers.

[14] Alhayek A., Syamsir A., Supian A.B.M., Usman F., Asyraf M.R.M., Atiqah M.A. (2022). Flexural Creep Behaviour of Pultruded GFRP Composites Cross-Arm: A Comparative Study on the Effects of Stacking Sequence. Polymers.

[15] Özkılıç Y.O., Gemi L., Madenci E., Aksoylu C. (2023). Effects of stirrup spacing on shear performance of hybrid composite beams produced by pultruded GFRP profile infilled with reinforced concrete. Arch. Civ. Mech. Eng..

[16] Gemi L., Madenci E., Özkılıç Y.O. (2021). Experimental, analytical and numerical investigation of pultruded GFRP composite beams infilled with hybrid FRP reinforced concrete. Eng. Struct..

[17] Madenci E., Özkılıç Y.O., Aksoylu C., Safonov A. (2022). The Effects of Eccentric Web Openings on the Compressive Performance of Pultruded GFRP Boxes Wrapped with GFRP and CFRP Sheets. Polymers.

[18] Wong S., Shanks R. (2008). Creep behaviour of biopolymers and modified flax fibre composites. Compos. Interfaces.

[19] Özkılıç Y.O., Gemi L., Madenci E., Aksoylu C., Kalkan İ. (2022). Effect of the GFRP wrapping on the shear and bending Behavior of RC beams with GFRP encasement. Struct. Eng. Mech..

[20] Bakar M.S.A., Mohamad D., Ishak Z.A.M., Yusof Z.M., Salwi N. (2018). Durability control of moisture degradation in GFRP cross arm transmission line towers. AIP Conf. Proc..

[21] Asyraf M.R.M., Ishak M.R., Syamsir A., Amir A.L., Nurazzi N.M., Norrrahim M.N.F., Asrofi M., Rafidah M., Ilyas R.A., Rashid M.Z.A. (2023). Filament-wound glass-fibre reinforced polymer composites: Potential applications for cross arm structure in transmission towers. Polym. Bull..

[22] Beddu S., Syamsir A., Arifin Z., Ishak M. Creep behavior of glass fibre reinforced polymer structures in crossarms transmission line towers. Proceedings of the AIP Conference Proceedings.

[23] Lu T., Solis-Ramos E., Yi Y., Kumosa M. (2018). UV degradation model for polymers and polymer matrix composites. Polym. Degrad. Stab..

[24] D’Antino T., Pisani M.A. (2019). Long-term behavior of GFRP reinforcing bars. Compos. Struct..

[25] Mahmood T., Kanapathipillai S., Chowdhury M. (2013). A model for creep life prediction of thin tube using strain energy density as a function of stress triaxiality under quasistatic loading employing elastic-creep & elastic-plastic-creep deformation. Front. Mech. Eng..

[26] Xu Y., Lee S.Y., Wu Q. (2011). Creep analysis of bamboo high-density polyethylene composites: Effect of interfacial treatment and fiber loading level. Polym. Compos..

[27] Asyraf M.R.M., Ishak M.R., Sapuan S.M., Yidris N. (2021). Comparison of Static and Long-term Creep Behaviors between Balau Wood and Glass Fiber Reinforced Polymer Composite for Cross-arm Application. Fibers Polym..

[28] Al Rashid A., Khalid M.Y., Imran R., Ali U., Koc M. (2020). Utilization of banana fiber-reinforced hybrid composites in the sports industry. Materials.

[29] Correia J., Cabral-Fonseca S. Durability of glass fibre reinforced polyester (GFRP) pultruded profiles used in civil engineering applications. Proceedings of the Composites in Construction 2005—Third International Conference.

[30] Zafar A., Bertocco F., Schjødt-Thomsen J., Rauhe J.C. (2012). Investigation of the long term effects of moisture on carbon fibre and epoxy matrix composites. Compos. Sci. Technol..

[31] Beloshenko V., Voznyak Y., Voznyak A., Savchenko B. (2017). New approach to production of fiber reinforced polymer hybrid composites. Compos. Part B Eng..

[32] Othman A., Abdullah S., Ariffin A.K., Mohamed N.A.N. (2014). Investigating the quasi-static axial crushing behavior of polymeric foam-filled composite pultrusion square tubes. J. Mater..

[33] Gemi L., Madenci E., Özkılıç Y.O., Yazman Ş., Safonov A. (2022). Effect of Fiber Wrapping on Bending Behavior of Reinforced Concrete Filled Pultruded GFRP Composite Hybrid Beams. Polymers.

[34] Mahmoud Zaghloul M.Y., Yousry Zaghloul M.M., Yousry Zaghloul M.M. (2022). Physical analysis and statistical investigation of tensile and fatigue behaviors of glass fiber-reinforced polyester via novel fibers arrangement. J. Compos. Mater..

[35] Zaghloul M.M.Y., Mohamed Y.S., El-Gamal H. (2019). Fatigue and tensile behaviors of fiber-reinforced thermosetting composites embedded with nanoparticles. J. Compos. Mater..

[36] Norrrahim M.N.F., Knight V.F., Nurazzi N.M., Jenol M.A., Misenan M.S.M., Janudin N., Kasim N.A.M., Shukor M.F.A., Ilyas R.A., Asyraf M.R.M. (2022). The Frontiers of Functionalized Nanocellulose-Based Composites and Their Application as Chemical Sensors. Polymers.

[37] Norizan M.N., Shazleen S.S., Alias A.H., Sabaruddin F.A., Asyraf M.R.M., Zainudin E.S., Abdullah N., Samsudin M.S., Kamarudin S.H., Norrrahim M.N.F. (2022). Nanocellulose-Based Nanocomposites for Sustainable Applications: A Review. Nanomaterials.

[38] Rahman I., Singh P., Dev N., Arif M., Yusufi F.N.K., Azam A., Alam M.M., Singh S., Chohan J.S., Kumar R. (2022). Improvements in the Engineering Properties of Cementitious Composites Using Nano-Sized Cement and Nano-Sized Additives. Materials.

[39] Patel P., Chokshi S. (2018). A Review of Fabrication Methods and Stacking Sequence Arrangements of Fiber for Composite. Int. J. Curr. Eng. Sci. Res..

[40] Abu Bakar M.S., Salit M.S., Mohamad Yusoff M.Z., Zainudin E.S., Ya H.H. (2020). The crashworthiness performance of stacking sequence on filament wound hybrid composite energy absorption tube subjected to quasi-static compression load. J. Mater. Res. Technol..

[41] Zakaria M.R., Md Akil H., Abdul Kudus M.H., Ullah F., Javed F., Nosbi N. (2019). Hybrid carbon fiber-carbon nanotubes reinforced polymer composites: A review. Compos. Part B Eng..

[42] Mir S.S., Hasan M., Hasan S.M.N., Hossain M.J., Nafsin N. (2017). Effect of Chemical Treatment on the Properties of Coir Fiber Reinforced Polypropylene and Polyethylene Composites. Polym. Compos..

[43] Nadhirah A., Mohamad D., Zainoodin M., Nabihah S., Mubin N., Itam Z., Mansor H., Kamal N.M., Muda Z.C., Nasional U.T. (2017). Properties of fiberglass crossarm in transmission tower—A review. Int. J. Appl. Eng. Res..

[44] Amir A.L., Ishak M.R., Yidris N., Zuhri M.Y.M., Asyraf M.R.M. (2021). Advances of composite cross arms with incorporation of material core structures: Manufacturability, recent progress and views. J. Mater. Res. Technol..

[45] Abd Halim S., Abu Bakar A.H., Illias H.A., Nor Hassan N.H., Mokhlis H., Terzija V. (2016). Lightning back flashover tripping patterns on a 275/132 kV quadruple circuit transmission line in Malaysia. IET Sci. Meas. Technol..

[46] Abd Rahman M.S., Ab Kadir M.Z.A., Ab-Rahman M.S., Osman M., Mohd Nor S.F., Mohd Zainuddin N. (2020). Effects of a Crossarm Brace Application on a 275 kV Fiberglass-Reinforced Polymer Crossarm Subjected to a Lightning Impulse. Energies.

[47] Asyraf M.R.M., Ishak M.R., Sapuan S.M., Yidris N. (2019). Conceptual design of creep testing rig for full-scale cross arm using TRIZ-Morphological chart-analytic network process technique. J. Mater. Res. Technol..

[48] Asyraf M.R.M., Ishak M.R., Sapuan S.M., Yidris N., Ilyas R.A., Rafidah M., Razman M.R. (2020). Evaluation of design and simulation of creep test rig for full-scale cross arm structure. Adv. Civ. Eng..

[49] Tian L., Pan H., Ma R., Zhang L., Liu Z. (2020). Full-scale test and numerical failure analysis of a latticed steel tubular transmission tower. Eng. Struct..

[50] An L., Wu J., Zhang Z., Zhang R. (2018). Failure analysis of a lattice transmission tower collapse due to the super typhoon Rammasun in July 2014 in Hainan Province, China. J. Wind Eng. Ind. Aerodyn..

[51] Zhu Y., Wang L., Yu J., Fang J. Optimal insulation design for new-type transmission tower with composite cross-arm. Proceedings of the International Symposium on Electrical Insulating Materials.

[52] Engineering Department of TNB Transmission Division (2013). Investigation Report on Wooden Crossarm Failure at 132kV KKSRPPAN L2.

[53] Yang X., Li N., Peng Z., Liao J., Wang Q. (2014). Potential distribution computation and structure optimization for composite cross-arms in 750 kV AC transmission line. IEEE Trans. Dielectr. Electr. Insul..

[54] Syamsir A., Mohamad D., Beddu S., Itam Z., Sadon S.N. (2019). A review on durability and degradation of glass fiber reinforced polymer structures. Int. J. Adv. Sci. Technol..

[55] Fairuz A.M., Sapuan S.M., Zainudin E.S., Jaafar C.N.A. (2014). Polymer composite manufacturing using a pultrusion process: A review. Am. J. Appl. Sci..

[56] Bhudolia S.K., Kam K.K.C., Joshi S.C. (2018). Mechanical and vibration response of insulated hybrid composites. J. Ind. Text..

[57] Asyraf M.R.M., Ishak M.R., Sapuan S.M., Yidris N., Rafidah M., Ilyas R.A., Razman M.R. (2020). Potential application of green composites for cross arm component in transmission tower: A brief review. Int. J. Polym. Sci..

[58] Rawi I.M., Rahman M.S.A., Ab Kadir M.Z.A., Izadi M. Wood and fiberglass crossarm performance against lightning strikes on transmission towers. Proceedings of the International Conference on Power Systems Transient (IPST).

[59] Peesapati V., Zachariades C., Li Q., Rowland S.M., Cotton I., Allison F., Chambers D., Densley J. (2012). 3D electric field computation of a composite cross-arm. Proceedings of the IEEE International Symposium on Electrical Insulation.

[60] Aksoylu C., Özkılıç Y.O., Madenci E., Safonov A. (2022). Compressive Behavior of Pultruded GFRP Boxes with Concentric Openings Strengthened by Different Composite Wrappings. Polymers.

[61] Asyraf M.R.M., Ishak M.R., Sapuan S.M., Yidris N. (2020). Conceptual design of multi-operation outdoor flexural creep test rig using hybrid concurrent engineering approach. J. Mater. Res. Technol..

[62] Itam Z., Ishak Z.A.M., Yusof Z.M., Salwi N., Zainoodin M. (2018). Effect on the temperature behavior of glass fiber reinforced polymer (GFRP) in various application—A review. AIP Conf. Proc..

[63] Syamsir A., Ishak Z.A.M., Yusof Z.M., Salwi N., Nadhirah A. (2018). Durability control of UV radiation in glass fiber reinforced polymer (GFRP)—A review. AIP Conf. Proc..

[64] Mohamad D., Syamsir A., Itam Z., Bakar H.A., Abas A., Ng F.C., Razali M.F., Seman S.A.H.A. (2019). Numerical Simulation on the Statics Deformation Study of Composite Cross Arms of Different Materials and Configurations. IOP Conf. Ser. Mater. Sci. Eng..

[65] Alhayek A., Syamsir A., Anggraini V., Muda Z.C., Nor N.M. (2019). Numerical Modelling of Glass Fiber Reinforced Polymer (GFRP) Cross Arm. Int. J. Recent Technol. Eng..

[66] Mohamad D., Syamsir A., Beddu S., Abas A., Ng F.C., Razali M.F., Seman S.A.H.A. (2019). Numerical Study of Composite Fiberglass Cross Arms under Statics Loading and Improvement with Sleeve Installation. IOP Conf. Ser. Mater. Sci. Eng..

[67] Moschiar S.M., Reboredo M.M., Kenny J.M., Vázquez A. (1996). Analysis of pultrusion processing of composites of unsaturated polyester resin with glass fibers. Polym. Compos..

[68] Faruk O., Bledzki A.K., Fink H.-P., Sain M. (2012). Biocomposites reinforced with natural fibers: 2000–2010. Prog. Polym. Sci..

[69] Lackey E., Vaughan J.G., Inamdar K., Hancock B. (2007). Statistical characterization of pultruded composites with natural fiber reinforcements—Part A: Fabrication. J. Nat. Fibers.

[70] Yeh H.Y., Yang S.C. (1997). Building of a composite transmission tower. J. Reinf. Plast. Compos..

[71] Baran I., Tutum C.C., Nielsen M.W., Hattel J.H. (2013). Process induced residual stresses and distortions in pultrusion. Compos. Part B Eng..

[72] Huchang W., Xueming W. (2011). Material Selection for Transmission Tower Made of Fiber Reinforced Plastics. Electr. Power Constr..

[73] Ibrahim S., Polyzois D., Hassan S.K. (2000). Development of glass fiber reinforced plastic poles for transmission and distribution lines. Can. J. Civ. Eng..

[74] Supian A.B.M., Sapuan S.M., Zuhri M.Y.M., Zainudin E.S., Ya H.H., Hisham H.N. (2021). Effect of winding orientation on energy absorption and failure modes of filament wound kenaf/glass fibre reinforced epoxy hybrid composite tubes under intermediate-velocity impact (IVI) load. J. Mater. Res. Technol..

[75] Asyraf M.R.M., Ishak M.R., Norrrahim M.N.F., Amir A.L., Nurazzi N.M., Ilyas R.A., Asrofi M., Rafidah M., Razman M.R. (2022). Potential of Flax Fiber Reinforced Biopolymer Composites for Cross-Arm Application in Transmission Tower: A Review. Fibers Polym..

[76] Asyraf M.R.M., Rafidah M., Ishak M.R., Sapuan S.M., Yidris N., Ilyas R.A., Razman M.R. (2020). Integration of TRIZ, Morphological Chart and ANP method for development of FRP composite portable fire extinguisher. Polym. Compos..

[77] Morozov E.V. (2006). The effect of filament-winding mosaic patterns on the strength of thin-walled composite shells. Compos. Struct..

[78] Lee C.S., Hwang W., Park H.C., Han K.S. (1999). Failure of carbon/epoxy composite tubes under combined axial and torsional loading 1. Experimental results and prediction of biaxial strength by the use of neural networks. Compos. Sci. Technol..

[79] Cardoso D.C.T., Togashi B.S. (2018). Experimental investigation on the flexural-torsional buckling behavior of pultruded GFRP angle columns. Thin Walled Struct..

[80] Hashim S.A., Nisar J.A. (2013). An investigation into failure and behaviour of GFRP pultrusion joints. Int. J. Adhes. Adhes..

[81] Syamsir A., Nor N.M., Zaidi A.M.A. (2012). Failure analysis of Carbon Fiber Reinforced Polymer (CFRP) bridge using composite material failure theories. Adv. Mater. Res..

[82] Sousa J.M., Correia J.R., Firmo J.P., Cabral-Fonseca S., Gonilha J. (2018). Effects of thermal cycles on adhesively bonded joints between pultruded GFRP adherends. Compos. Struct..

[83] Mohamad D., Syamsir A., Sa’don S.N., Zahari N.M., Seman S.A.H.A., Razali M.F., Abas A., Ng F.C. (2019). Stacking sequence effects on performance of composite laminate structure subjected to multi-axial quasi-static loading stacking sequence. IOP Conf. Ser. Mater. Sci. Eng..

[84] Mohamad D., Syamsir A., Beddu S., Kamal N.L.M., Zainoodin M.M., Razali M.F., Abas A., Seman S.A.H.A., Ng F.C. (2019). Effect of laminate properties on the failure of cross arm structure under multi-axial load. IOP Conf. Ser. Mater. Sci. Eng..

[85] Turon A., Costa J., Camanho P.P., Dávila C.G. (2007). Simulation of delamination in composites under high-cycle fatigue. Compos. Part A Appl. Sci. Manuf..

[86] Cardoso D.C.T., Harries K.A., Batista E.D.M. (2014). Compressive strength equation for GFRP square tube columns. Compos. Part B Eng..

[87] Li H., Yao Y., Guo L., Zhang Q., Wang B. (2018). The effects of delamination deficiencies on compressive mechanical properties of reinforced composite skin structures. Compos. Part B Eng..

[88] Vahid Movahedi-Rad A., Anastasios P. (2019). Vassilopoulos; Thomas Keller Creep effects on tension-tension fatigue behavior of angle-ply GFRP composite laminates. Int. J. Fatigue.

[89] Johari A.N., Ishak M.R., Leman Z., Yusoff M.Z.M., Asyraf M.R.M. (2020). Creep behaviour monitoring of short-term duration for fiber-glass reinforced composite cross-arms with unsaturated polyester resin samples using conventional analysis. J. Mech. Eng. Sci..

[90] Asyraf M.R.M., Ishak M.R., Sapuan S.M., Yidris N. (2021). Influence of Additional Bracing Arms as Reinforcement Members in Wooden Timber Cross-Arms on Their Long-Term Creep Responses and Properties. Appl. Sci..

[91] Wang J., Yang N., Zhao J., Wang D., Wang Y., Li K., He Z., Wang B. (2016). Design and experimental verification of composite impact attenuator for racing vehicles. Compos. Struct..

[92] Qiao P., Yang M., Bobaru F. (2008). Impact mechanics and high-energy absorbing materials: Review. J. Aerosp. Eng..

[93] Syamsir A., Amat A.H., Usman F., Itam Z., Kamal N.L.M., Zahari N.M., Chairi M., Imani R. (2021). Effect of fiber orientation on ultimate tensile strength and Young’s modulus of fabricated glass fiber reinforced polymer plates. AIP Conf. Proc..

[94] Johari A.N., Ishak M.R., Leman Z., Yusoff M.Z.M., Asyraf M.R.M. (2020). Influence of CaCO_3_ in pultruded glass fiber/unsaturated polyester resin composite on flexural creep behavior using conventional and time-temperature superposition principle methods. Polimery/Polymers.

[95] Grzybowski S., Disyadej T. Electrical performance of fiberglass crossarm in distribution and transmission lines. Proceedings of the 2008 IEEE/PES Transmission and Distribution Conference and Exposition.

[96] Babaee M., Jonoobi M., Hamzeh Y., Ashori A. (2015). Biodegradability and mechanical properties of reinforced starch nanocomposites using cellulose nanofibers. Carbohydr. Polym..

[97] Angelov I., Wiedmer S., Evstatiev M., Friedrich K., Mennig G. (2007). Pultrusion of a flax/polypropylene yarn. Compos. Part A Appl. Sci. Manuf..

[98] Du Y., Yan N., Kortschot M.T. (2013). An experimental study of creep behavior of lightweight natural fiber-reinforced polymer composite/honeycomb core sandwich panels. Compos. Struct..

[99] Couceiro I., París J., Martínez S., Colominas I., Navarrina F., Casteleiro M. (2016). Structural optimization of lattice steel transmission towers. Eng. Struct..

[100] Turvey G.J., Zhang Y. (2018). Mechanical properties of pultruded GFRP WF, channel and angle profiles for limit state/permissible stress design. Compos. Part B Eng..

[101] Sharaf H.K., Ishak M.R., Sapuan S.M., Yidris N., Fattahi A. (2020). Experimental and numerical investigation of the mechanical behavior of full-scale wooden cross arm in the transmission towers in terms of load-deflection test. J. Mater. Res. Technol..

[102] Abd Malek N.J., Hassan R., Ali E.M., Shakimon M.N. (2016). Double shear test behaviour of balau species for different end distance. J. Teknol..

[103] Henriksson M., Henriksson G., Berglund L.A., Lindström T. (2007). An environmentally friendly method for enzyme-assisted preparation of microfibrillated cellulose (MFC) nanofibers. Eur. Polym. J..

[104] Kanyilmaz A. (2017). Role of compression diagonals in concentrically braced frames in moderate seismicity: A full scale experimental study. J. Constr. Steel Res..

[105] Gokdemir H., Ozbasaran H., Dogan M., Unluoglu E., Albayrak U. (2013). Effects of torsional irregularity to structures during earthquakes. Eng. Fail. Anal..

[106] Patil D.M., Sangle K.K. (2015). Seismic behaviour of different bracing systems in high rise 2-D steel buildings. Structures.

[107] Chang E., Dover W.D. (2001). Characteristic parameters for stress distribution along the intersection of tubular Y, T, X and DT joints. J. Strain Anal. Eng. Des..

[108] Guo Y.L., Fu P.P., Zhou P., Tong J.Z. (2016). Elastic buckling and load resistance of a single cross-arm pre-tensioned cable stayed buckling-restrained brace. Eng. Struct..

[109] Klasson A., Crocetti R., Hansson E.F. (2016). Slender steel columns: How they are affected by imperfections and bracing stiffness. Structures.

[110] Sharaf H.K., Ishak M.R., Sapuan S.M., Yidris N. (2020). Conceptual design of the cross-arm for the application in the transmission towers by using TRIZ–morphological chart–ANP methods. J. Mater. Res. Technol..

[111] Qin Q., Wang T.J. (2013). Low-velocity impact response of fully clamped metal foam core sandwich beam incorporating local denting effect. Compos. Struct..

[112] Zhang L., Liu W., Wang L., Ling Z. (2019). Mechanical behavior and damage monitoring of pultruded wood-cored GFRP sandwich components. Compos. Struct..

[113] Mishra G., Mohapatra S.R., Behera P.R., Dash B., Mohanty U.K., Ray B.C. (2010). Environmental stability of GFRP laminated composites: An emphasis on mechanical behaviour. Aircr. Eng. Aerosp. Technol..

[114] Alessi S., Pitarresi G., Spadaro G. (2014). Effect of hydrothermal ageing on the thermal and delamination fracture behaviour of CFRP composites. Compos. Part B Eng..

[115] Khan L.A., Mahmood A.H., Syed A.S., Khan Z.M., Day R.J. (2012). Effect of hygrothermal conditioning on the fracture toughness of carbon/epoxy composites cured in autoclave/Quickstep. J. Reinf. Plast. Compos..

